# Gremlin-1 for the Differential Diagnosis of Idiopathic Pulmonary Fibrosis Versus Other Interstitial Lung Diseases: A Clinical and Pathophysiological Analysis

**DOI:** 10.1007/s00408-021-00440-y

**Published:** 2021-03-26

**Authors:** Yoichiro Aoshima, Yasunori Enomoto, Shigeki Muto, Shiori Meguro, Hideya Kawasaki, Isao Kosugi, Tomoyuki Fujisawa, Noriyuki Enomoto, Naoki Inui, Yutaro Nakamura, Takafumi Suda, Toshihide Iwashita

**Affiliations:** 1 Department of Regenerative and Infectious Pathology, Hamamatsu University School of Medicine, 1-20-1 Handayama, Hamamatsu, Shizuoka 431-3192 Japan; 2 Second Division, Department of Internal Medicine, Hamamatsu University School of Medicine, Hamamatsu, Shizuoka Japan; 3 Department of Health Care, Seirei Center for Health Promotion and Preventive Medicine, Hamamatsu, Shizuoka Japan

**Keywords:** Gremlin-1, Interstitial lung disease, Idiopathic pulmonary fibrosis, Biomarker, Differential diagnosis

## Abstract

**Purpose:**

The differential diagnosis of interstitial lung diseases (ILDs), particularly idiopathic pulmonary fibrosis (IPF) versus other non-IPF ILDs, is important for selecting the appropriate treatment. This retrospective study aimed to explore the utility of gremlin-1 for the differential diagnosis.

**Methods:**

Serum gremlin-1 concentrations were measured using an ELISA in 50 patients with IPF, 42 patients with non-IPF ILD, and 30 healthy controls. The baseline clinical data, including pulmonary functions, prognosis, and three serum biomarkers (Krebs von den Lungen-6 [KL6], surfactant protein-D [SP-D], and lactate dehydrogenase [LDH]), were obtained through a medical record review for analyzing their associations with serum gremlin-1 concentrations. To evaluate the origin of gremlin-1, we performed immunostaining on lung sections.

**Results:**

Serum gremlin-1 concentrations were significantly higher in patients with IPF (mean concentration, 14.4 ng/mL), followed by those with non-IPF ILD (8.8 ng/mL) and healthy controls (1.6 ng/mL). The area under the curve for IPF versus non-IPF ILDs was 0.759 (95% confidence interval, 0.661–0.857), which was superior to that of KL6/SP-D/LDH. The sensitivity and specificity for gremlin-1 (cutoff, 10.4 ng/mL) was 72 and 69%, respectively. By contrast, serum gremlin-1 concentrations were not associated with the pulmonary functions nor the prognosis in all patients with ILDs. In immunostaining, the gremlin-1 was broadly upregulated in IPF lungs, particularly at myofibroblasts, bronchiolar/alveolar epithelium, and CD163-positive M2-like macrophages.

**Conclusions:**

Gremlin-1 may be a useful biomarker to improve the diagnostic accuracy for IPF compared to non-IPF ILDs, suggesting a role of this molecule in the pathogenesis of IPF.

## Introduction

Interstitial lung disease (ILD) is a heterogeneous disease in terms of its variety, particularly in regard to its clinical course and treatment [1]. Among ILDs, idiopathic pulmonary fibrosis (IPF) has a poor prognosis and requires a unique treatment strategy using antifibrotic drugs, while immunosuppressive therapy, which is frequently administered for non-IPF ILDs, is not recommended for IPF [2]. Accurately diagnosing IPF is therefore an important task for respiratory physicians.

The diagnosis of IPF requires evidence of a typical “usual interstitial pneumonia (UIP)” pattern either through radiology or pathology, as well as exclusion of the possibility of other non-IPF ILDs [2]. However, accurately diagnosing IPF can sometimes be difficult because non-IPF ILDs, including collagen vascular disease-related ILD and other fibrotic ILDs, can exhibit a UIP-like pattern [3–5]. Even for experts in ILD, diagnosing IPF with a high certainty is challenging and is an active research topic [6, 7]. In this context, biomarkers that can easily distinguish IPF from non-IPF ILDs would be helpful. In Japan, Krebs von den Lungen-6 (KL6) is one of the most popular biomarkers for the clinical management of ILDs. However, as several researchers have pointed out, this molecule is more suitable for evaluating disease behavior and prognosis than differentiating ILDs [8, 9], which suggests a need for another biomarker for the purpose of differential diagnosis.

We recently performed a microarray analysis using bleomycin-induced myofibroblasts and steady-state fibroblasts directly isolated from mouse lungs [10]. In the transcriptome data, interestingly, we found that gremlin-1, a secreted glycoprotein and an antagonist of bone morphogenetic protein-4 (BMP4), was significantly upregulated in the myofibroblasts of the fibrotic lungs. Consistent with our data, previous studies have also suggested that gremlin-1 is upregulated in human lungs with ILDs, particularly IPF [11, 12]. Based on these results, we therefore sought to explore the possibility of serum gremlin-1 as a novel biomarker for ILD.

## Materials and Methods

### Mouse Microarray Data

The detailed methods for the experiments using mouse samples have been described in one of our previous articles [10]. Briefly, lung myofibroblasts and steady-state fibroblasts were isolated by fluorescence-activated cell sorting in a CD49e^+^Sca-1^−^lineage (CD45/TER119/CD31/CD324/Lyve-1/CD146)^−^ population from bleomycin-treated mouse fibrotic lungs and in a PDGFRα^+^lineage^−^ population from normal untreated mouse lungs, respectively. We evaluated the transcriptome data of these cells by microarray analysis (Accession Number, GSE111043). Gene expression profiling with RNA amplification was performed by Takara Bio, Japan.

### Selection of Patients with ILD and Healthy Controls

**Fig. 1:**
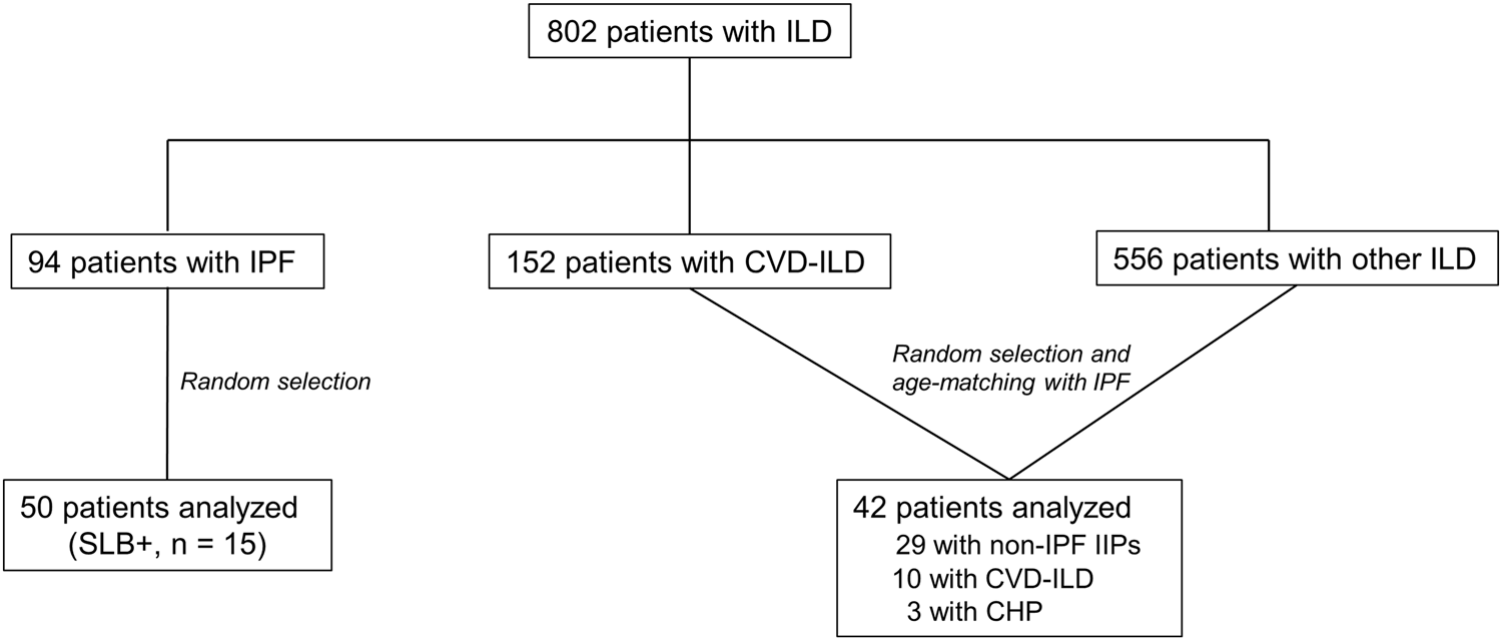
Flow chart of patient selection process. *CHP* chronic hypersensitivity pneumonitis, *CVD* collagen vascular disease, *IIP* idiopathic interstitial pneumonia, *ILD* interstitial lung disease, *IPF* idiopathic pulmonary fibrosis, *SLB* surgical lung biopsy

A database review between 2000 and 2017 at Hamamatsu University Hospital identified 50 randomly-selected patients with IPF and 42 age-matched patients with non-IPF ILD, including idiopathic interstitial pneumonias other than IPF (29 patients; 18 were unclassifiable, 10 had pathologically proven nonspecific interstitial pneumonia [NSIP], and 1 had pathologically proven pleuroparenchymal fibroelastosis), collagen vascular disease-associated ILD (10 patients; 3 had dermatomyositis, 3 had primary Sjögren’s syndrome, 2 had systemic scleroderma, 1 had rheumatoid arthritis, and 1 had microscopic polyangiitis), and chronic hypersensitivity pneumonitis (3 patients) (Fig. 1). Each ILD diagnosis was retrospectively re-confirmed for this study.

The patients’ medical records were reviewed to obtain the following clinical data at the time of serum sampling: sex, age, percent predicted forced vital capacity (%FVC), percent predicted diffusion capacity for carbon monoxide (%DLCO), partial pressure of arterial oxygen (PaO_2_) on room air, the disease extent on chest high-resolution computed tomography (HRCT), the radiological UIP compatibility (UIP; probable UIP; indeterminate for UIP; alternative diagnosis) on chest HRCT [2], and 3 serum biomarkers available in our clinical setting (KL6, surfactant protein-D [SP-D], and lactate dehydrogenase [LDH]). As controls, we recruited 30 age-matched healthy volunteers with no lung disease at the Seirei Center for Health Promotion and Preventive Medicine. The study was approved by the Institutional Review Board of Hamamatsu University School of Medicine (Approval Numbers: 14-365, 15-197, and 18-126) and by the Seirei Center (Approval Number: 30-08).

### Serum Sampling and Measurement of Gremlin-1 Concentrations

We obtained serum samples from the healthy volunteers during the health check-ups and from the patients with ILD at the time of the ILD diagnosis. Each participant agreed to undergo the procedure by providing their written informed consent. Serum gremlin-1 concentrations were quantified using sandwich enzyme-linked immunosorbent assay based on the manufacturer’s protocol (LifeSpan BioSciences, USA; #LS-F6538).

### Immunostaining

We evaluated formalin-fixed paraffin-embedded lung specimens from 3 patients with IPF, 5 patients with non-IPF ILD (1 patient with idiopathic fibrotic NSIP, 1 with dermatomyositis-associated f-NSIP, 1 with systemic scleroderma-associated f-NSIP, 1 with primary Sjögren's syndrome-associated UIP, and 1 with chronic hypersensitivity pneumonitis), and 3 healthy controls. Deparaffinized Sections (4-μm-thick) were preheated at 105 °C for 15 min (pH 9).

#### Immunohistochemistry

After inactivating endogenous peroxidase with 0.3% H_2_O_2_ for 10 min, the sections were incubated with a blocking/permeabilizing solution (10% goat serum in phosphate buffered saline and 0.1% Triton X-100 in phosphate buffered saline) for 60 min and then incubated with anti-gremlin-1 antibody (Bioss antibodies, USA; #bs-1475R. 1:100) at 4 °C overnight. The next day, the lung sections were incubated with peroxidase-labeling goat anti-rabbit IgG antibody (Nichirei Biosciences, Japan; #424131) for 60 min. The immunoreaction was visualized using 3,3-diaminobenzidine (Dako, USA: #K500711-2) and then counterstained with hematoxylin.

#### Immunofluorescence

After incubating with a blocking/permeabilizing solution for 60 min and then incubated with primary antibodies at 4 °C overnight (anti-gremlin-1 antibody [1:100], anti-αSMA antibody as a myofibroblast marker [DAKO/Agilent Technologies, USA; #GA61161-2. 1:200], anti-E-cadherin antibody [DAKO/Agilent Technologies, USA: #GA05961-2. 1:200] as an epithelial cell marker, and anti-CD163 antibody as an M2-like macrophage marker [Leica Biosystems, USA; #CD163-L-CE. 1:200]). The sections were then incubated with Hoechst 33342 (Sigma-Aldrich, USA; #14533. 1:1000) with Alexa Fluor conjugated secondary antibodies (Invitrogen, USA; All 1:500) at room temperature for 60 min.

### Statistical Analyses

We performed statistical analyses using EZR software (ver. 1.41). Coefficient values between each parameter were evaluated using Spearman rank correlation coefficient method. Group comparisons were performed using Fisher’s exact test, Mann–Whitney U test, or Kruskal–Wallis test with post-hoc Bonferroni test as appropriate. Multivariate analyses were done using a logistic regression model. In the analyses of receiver operating characteristic curves, area under the curve (AUC) values were compared using DeLong test. Cox proportional hazards model was used for a prognostic analysis. A *p* < 0.05 was considered statistically significant.

## Results

### Gene Expression of Gremlin-1

**Fig. 2:**
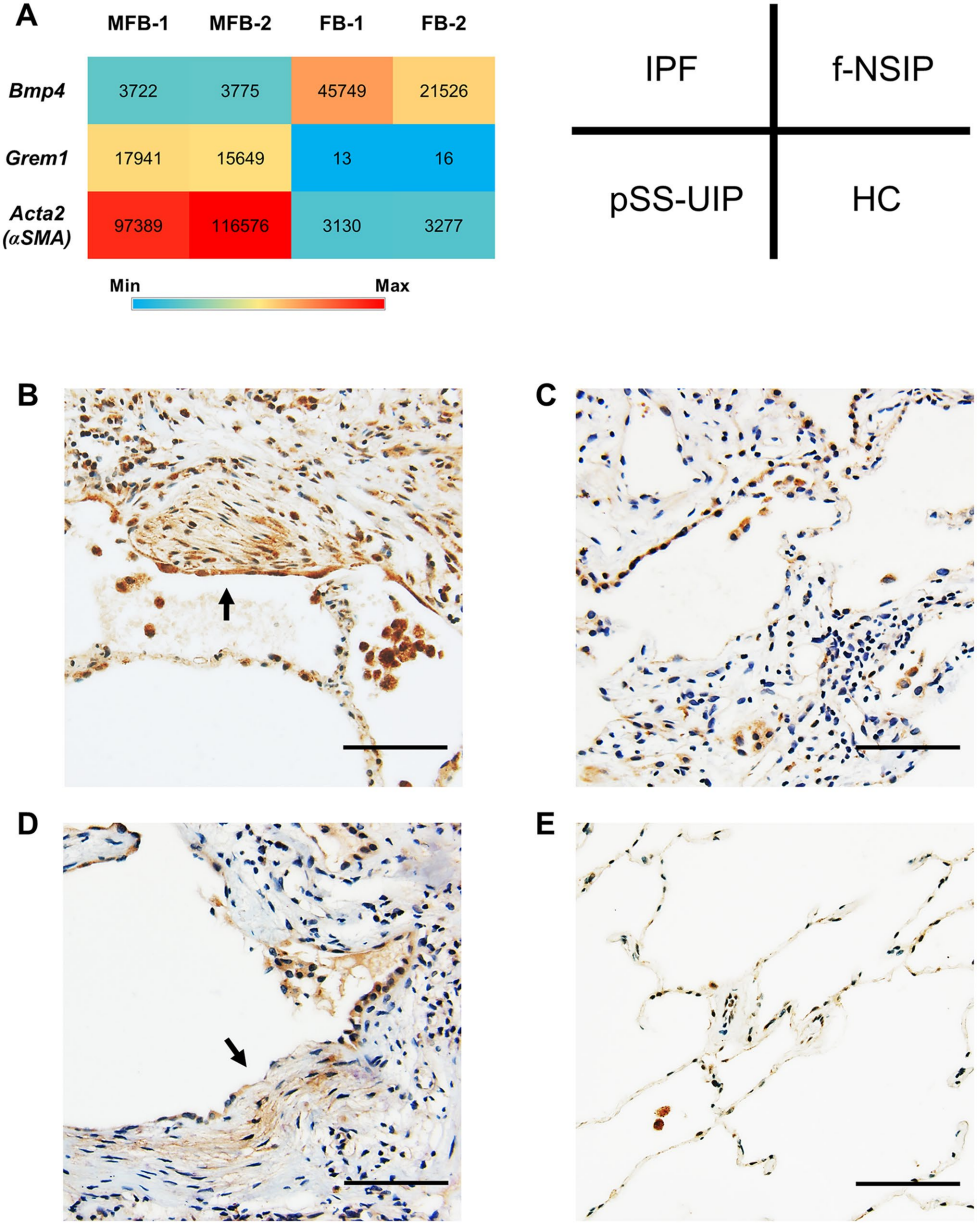
Gremlin-1 expression in mouse lung (myo)fibroblasts and human lungs. **a** mRNA signal comparisons between myofibroblasts (MFB) and steady-state fibroblasts (FB) by microarray analysis (*n* = 2 each). **b** Gremlin-1 protein expression in idiopathic pulmonary fibrosis (IPF). The arrow indicates fibroblastic focus. (patient profile: 68-year-old male; serum gremlin-1 concentration = 26.3 ng/mL). **c** Gremlin-1 protein expression in idiopathic fibrotic nonspecific interstitial pneumonia (f-NSIP). (patient profile: 49-year-old male; serum gremlin-1 concentration = 2.3 ng/mL). **d** Gremlin-1 protein expression in primary Sjögren's syndrome-associated usual interstitial pneumonia (pSS-UIP). The arrow indicates fibroblastic focus (patient profile: 74-year-old male; serum gremlin-1 concentration = 13.2 ng/mL). **e** Gremlin-1 protein expression in healthy control (HC) lung (original magnification: ×200; Scale bar: 100 μm)

According to our published mouse microarray dataset (GSE111043), gremlin-1 mRNA (*Grem1*) was significantly upregulated, while BMP4 mRNA was downregulated in bleomycin-induced lung myofibroblasts compared with steady-state fibroblasts (Fig. 2a), which is consistent with previous human data comparing IPF (myo) fibroblasts with normal fibroblasts [11]. The signal ratio of *Grem1* was similar to that of αSMA (*Acta2*) mRNA, a canonical marker of myofibroblasts. In contrast, the mRNA expression of other BMP antagonists, including noggin and chordin, was not significantly different between the cells.

### Protein Expression of Gremlin-1

**Fig. 3:**
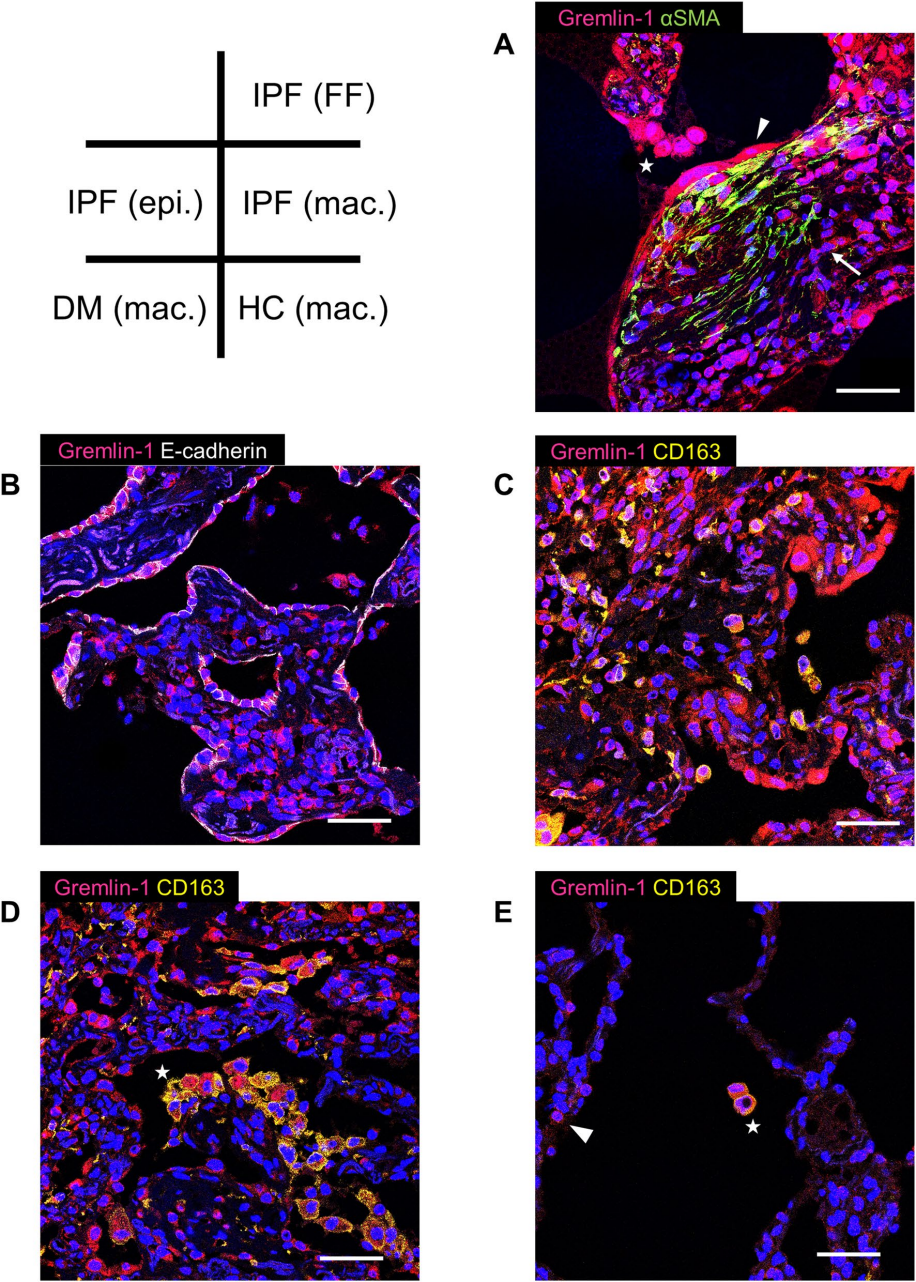
Immunofluorescence in human lungs. **a** Fibroblastic focus (FF) in idiopathic pulmonary fibrosis (IPF). Gremlin-1 (*red*) is co-localized at αSMA (*green*)-positive myofibroblasts in the FF, as well as the covering epithelium (*triangle*), alveolar macrophages (*star*), and αSMA-negative fibroblast-like cells (*arrow*). **b** Epithelium in IPF. Gremlin-1 (*red*) is typically upregulated in E-cadherin (*white*)-positive epithelial cells. **c** Macrophages in IPF. Gremlin-1 (*red*) is also found at CD163 (*yellow*)-positive macrophages in the alveolus and interstitium. **d** Macrophages in dermatomyositis (DM)-associated interstitial lung disease. Gremlin-1 (*red*) upregulation is evident at gathered CD163 (*yellow*)-positive alveolar macrophages (*star*) and epithelium. **e** Macrophages in healthy control (HC) lung. Weak positivity of gremlin-1 (*red*) can be seen in CD163 (*yellow*)-positive alveolar macrophages (*star*) and a part of epithelial cells (*triangle*) (original magnification: ×630; Scale bar: 40 μm)

To determine the origin of gremlin-1 in human lungs, we performed immunostaining. In IPF lung sections, gremlin-1 protein was broadly expressed in the fibrotic interstitium. Consistent with the mRNA results, the upregulation was particularly apparent in αSMA-positive myofibroblasts, regardless of whether they formed fibroblastic foci (Figs. 2b and 3a). Smooth muscle hyperplasia and αSMA-negative fibroblast-like cells also showed gremlin-1 positivity, although not consistently. In addition to those mesenchymal cells, gremlin-1 upregulation was commonly found in bronchiolar/alveolar epithelium (Fig. 3b) and CD163-positive M2-like macrophages both in the alveolus and interstitium (Fig. 3c). In non-IPF ILDs, gremlin-1 deposition in the interstitium was mild but occurred in the fibroblastic foci, epithelium, and macrophages (Figs. 2c, d and 3d). The number of αSMA-positive myofibroblasts or fibroblastic foci was typically lower in non-IPF ILDs than in IPF. In dermatomyositis-associated ILD lung sections, the infiltration of CD163-positive macrophages was severe, and these cells typically expressed gremlin-1 (Fig. 3d). In normal lung sections, in contrast, gremlin-1 expression was totally weak but was found in the alveolar macrophages and part of the epithelium, compared to negative-control sections (Figs. 2e and 3e).

### Clinical Data and Biomarker Analysis

Table 1 summarizes our cohort’s baseline patient characteristics. As expected, patients with all-type ILD exhibited lower %FVC and higher serum LDH concentrations than the healthy controls. When compared with those with non-IPF ILD, patients with IPF were significantly more male-dominant and showed relatively lower %FVC. The majority of HRCT pattern of patients with IPF was UIP pattern and the extent of abnormal shadows was significantly larger than patients with non-IPF ILD.

**Table 1:** Comparison of baseline characteristics between healthy controls, patients with IPF, and patients with non-IPF ILD

	Healthy controls (*n* = 30)	Patients with ILDs		CV with gremlin-1 in ILDs (*p* value)	*P* values between IPF and non-IPF	
		IPF (*n* = 50)	Non-IPF (*n* = 42)		Univariate	Multivariate
Serum gremlin-1 (ng/mL)	1.5 (1.2–1.9)	11.5 (10.1–17.6)	9.1 (7.0–11.5)	–	< 0.01	0.02
Male	25 (83.3%)	44 (88.0%)	26 (61.9%)	*r* = − 0.27 (*p* = 0.01)	< 0.01	0.65
Age, years	65.5 (61.0–70.8)	68.0 (63.0–75.8)	67.0 (61.3–72.8)	*r* = 0.099 (*p* = 0.35)	0.18	–
% Predicted FVC (%)	98.3 (90.9–107.7)	63.3 (53.9–86.2)	77.3 (68.3–87.3)	*r* = − 0.15 (*p* = 0.19)	0.09	0.59
% Predicted DLCO (%)	NE	62.4 (49.2–84.7)	72.8 (62.1–83.4)	*r* = 0.02 (*p* = 0.87)	0.14	–
PaO_2_ (Torr on room air)	NE	75.9 (69.0–86.0)	77.8 (71.3–82.0)	*r* = − 0.19 (*p* = 0.07)	0.55	–
Serum LDH (U/L)	179.5 (154.8–196.5)	249.0 (215.3–283.0)	240.5 (188.8–269.0)	*r* = 0.30 (*p* = 0.0038)	0.27	–
Serum KL6 (U/mL)	NE	1087.5 (775.3–1528.5)	1035.5 (741.3–1652.8)	*r* = 0.20 (*p* = 0.065)	0.90	–
Serum SP-D (ng/mL)	NE	216.5 (155.5–330.3)	191.0 (128.0–270.0)	*r *= 0.21 (*p* = 0.052)	0.15	–
UIP compatibility on chest HRCT^a^: UIP/probable/indeterminate/alternative	NE	31/14/5/0	0/15/12/15	*r* = 0.40 (*p* < 0.01)	< 0.01	< 0.01
Disease extent on chest HRCT, grade 1–4^b^	NE	3 (2–3)	2 (2–2)	*r* = 0.16 (*p* = 0.12)	< 0.01	0.19

Data are described as *n* (%) or median (interquartile range). CVs and subsequent *p *values are evaluated by Spearman’s rank correlation coefficient method. For group comparisons between patients with IPF and those with non-IPF ILD, *p* values in univariate analyses are calculated using Fisher’s exact test or Mann–Whitney U test as appropriate. Multivariate logistic regression analyses are performed using factors with *p* < 0.1 in the univariate analyses

*CV* coefficient value, *DLCO* diffusion capacity for carbon monoxide, *FVC* forced vital capacity, *HRCT* high-resolution computed tomography, *ILD* interstitial lung disease, *IPF* idiopathic pulmonary fibrosis, *KL6* Krebs von den Lungen-6, *LDH* lactate dehydrogenase, *NE* not evaluated, *PaO*_*2*_ partial pressure of arterial oxygen, *SP-D* surfactant protein-D, *UIP* usual interstitial pneumonia

^a^Classification of imaging patterns based on the recent international criteria [2]

^b^Area of abnormal shadows in total lungs: grade 1 =  < 10%; 2 = 10–25%; 3 = 25–50%; 4 =  > 50%

**Fig. 4:**
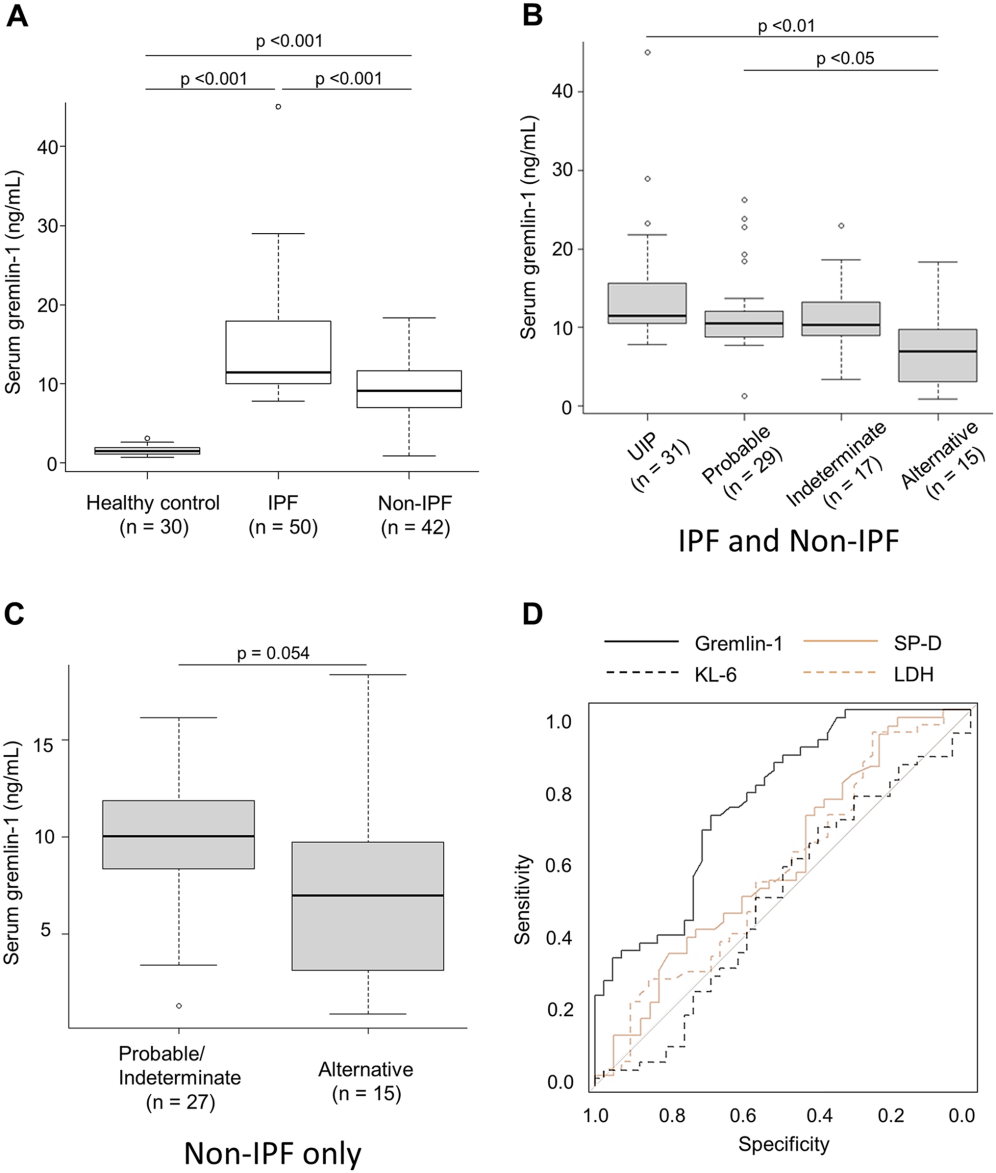
Serum gremlin-1 concentrations in patients with interstitial lung disease (ILD). **a** Comparison of serum gremlin-1 concentrations between patients with idiopathic pulmonary fibrosis (IPF), patients with non-IPF ILD, and healthy controls. Statistical analysis is performed using Kruskal–Wallis test with post-hoc Bonferroni test. **b** Comparison of serum gremlin-1 concentrations with imaging patterns on chest high-resolution computed tomography (usual interstitial pneumonia [UIP]; probable UIP; indeterminate for UIP; alternative diagnosis) among patients with IPF and non-IPF ILD. Statistical analysis is performed using Kruskal–Wallis test with post-hoc Bonferroni test. **c** Comparison of serum gremlin-1 concentrations with imaging patterns on chest high-resolution computed tomography among patients with non-IPF ILD only. Statistical analysis is performed using Mann–Whitney U test. **d** Receiver operating characteristic curves to distinguish patients with IPF from those with non-IPF ILD using gremlin-1, Krebs von den Lungen-6 (KL6), surfactant protein-D (SP-D), and lactate dehydrogenase (LDH)

As shown in Table 1 and Fig. 4a, serum gremlin-1 concentrations were significantly higher in patients with IPF (mean concentration, 14.4 ng/mL; median, 11.5; interquartile range, 10.1–17.6), followed by those with non-IPF ILD (mean, 8.8 ng/mL; median, 9.1; interquartile range, 7.0–11.5) and healthy controls (mean, 1.6 ng/mL; median, 1.5; interquartile range, 1.2–1.9). Consistent with that, among patients with all-type ILD, those with UIP pattern or “probable UIP” pattern on chest HRCT had higher serum gremlin-1 concentrations than patients with “alternative diagnosis” pattern (Fig. 4b). Interestingly, even in non-IPF ILDs, the higher UIP compatibility on chest HRCT showed a tendency for a more increase in serum gremlin-1 (Fig. 4c). However, the increase of serum gremlin-1 was independently associated with an IPF diagnosis in our multivariate logistic regression analysis including the factor of UIP compatibility (Table 1). In contrast, unexpectedly, we found no significant correlations of serum gremlin-1 concentrations with %FVC, %DLCO, PaO_2_, and the disease extent on chest HRCT. Additionally, serum gremlin-1 concentrations were not associated with the prognosis in patients with all-type ILD (hazard ratio per 1 ng/mL of gremlin-1 increase, 1.03; 95% confidence interval [CI] 0.99–1.07; *p* = 0.11), suggesting that this molecule would be not useful as a prognostic biomarker.

To evaluate the potential of gremlin-1 as a biomarker for the differential diagnosis of ILDs, we constructed receiver operating characteristic curves between IPF and non-IPF ILDs (Fig. 4d). The AUC of gremlin-1 was 0.759 (95% CI 0.661–0.857), which was significantly higher compared with the other 3 serum biomarkers: KL6 (AUC, 0.492; 95% CI 0.369–0.615; *p* < 0.001 vs. gremlin-1), SP-D (AUC, 0.591; 95% CI 0.470–0.712; *p* = 0.03 vs. gremlin-1), and LDH (AUC, 0.567; 95% CI 0.447–0.687; *p* = 0.004 vs. gremlin-1). Although we explored the possibility of these 3 biomarkers in combination with gremlin-1, none of them could not significantly increase the AUC value, compared to that of gremlin-1 alone. When the gremlin-1 cutoff was set at 10.4 ng/mL, the sensitivity and specificity for discriminating IPF from non-IPF ILD was 72 and 69%, respectively.

## Discussion

In this study, we demonstrated that the protein of gremlin-1 was upregulated in fibrotic lungs, particularly at myofibroblasts, CD163-positive M2-like macrophages, and bronchiolar/alveolar epithelium in IPF lungs. The serum gremlin-1 concentration was significantly higher in patients with IPF than those with non-IPF ILD, suggesting a pathophysiological role of this molecule in IPF.

Gremlin-1 is an endogenous BMP4 antagonist, and the antagonizing regulation employs extracellular and intracellular pathways [13]. This molecule is essential during respiratory development for normal airway patterning and for the differentiation of distal epithelial cells [14]. In contrast, gremlin-1 has been studied about the associations with abnormal lung conditions, such as hypoxia, pulmonary hypertension, and above all, lung fibrosis [13]. In fact, in our mouse microarray dataset, gremlin-1 was upregulated while BMP4 was downregulated in the myofibroblasts of fibrotic mouse lungs (Fig. 2a). The similar expression pattern of gremlin-1 and BMP4 is reproduced even in IPF lung-derived (myo)fibroblasts [11], implying a common role for myofibroblasts in fibrotic lungs in inhibiting BMP4 signaling, probably via gremlin-1 autocrine activity. BMP4 is thought to inhibit fibroblast proliferation and differentiation into myofibroblasts [11, 15]. In fact, a previous experimental study using noggin-insufficient mice revealed that the upregulation of BMP signaling could reduce the severity of bleomycin-induced lung fibrosis [16]. Thus, gremlin-1 upregulation and BMP signaling downregulation in myofibroblasts would support their own presence, leading to the persistence of lung fibrosis.

Based on our immunostaining results, the origin of lung gremlin-1 appears to be not only myofibroblasts but also the bronchiolar/alveolar epithelium and CD163-positive M2-like macrophages. Consistent with these results, a recent bulk RNA-seq data analysis of human cells also showed that *GREM1* mRNA expression in fibrotic lungs is significantly upregulated, even in alveolar type 2 epithelial cells (approximately 14.7-fold) and alveolar macrophages (approximately as much as 116.2-fold) compared with normal control cells [17]. Another study showed that gremlin-1 overexpression in lung epithelial cells induced mRNA upregulation of transforming growth factor-β1 (TGF-β1), a potent fibrosis-promoting factor. In fact, epithelial gremlin-1 overexpression could lead to lung fibrosis, even in vivo [18]. In contrast, it is well known that M2-like macrophages also play an important role during fibrogenesis, particularly as a primary source of TGF-β [19]. Gremlin-1 secretion may be an additional role for M2-like macrophages and might further activate the surrounding fibroblasts. Collectively, gremlin-1 upregulation in epithelium and macrophages could cooperatively contribute to the pathogenesis of lung fibrosis.

The increase in serum gremlin-1 concentration was significantly prominent in IPF compared with non-IPF ILDs. Although the exact mechanism underlying this gap is unclear, it could be mostly due to the difference in the quantity of myofibroblasts or fibroblastic foci, which are significantly more numerous in IPF than in non-IPF ILD lungs or in UIP pattern than in non-UIP pattern [4, 20, 21]. This may partly explain our finding that UIP compatibility on chest HRCT was associated with serum gremlin-1 concentrations, particularly even in patients with non-IPF ILD (Fig. 4b, c). In addition, based on our pathology findings, the severity of M2-like or M2-polarized macrophage infiltration might affect the serum gremlin-1 value. Compared with normal lungs, the significantly greater infiltration of M2-like macrophages has been reported in IPF lungs and in NSIP lungs [22]. As we have also suggested, M2-like macrophages are increased in the lungs of dermatomyositis-associated ILD [23]. Actually, our patients with dermatomyositis-associated-ILD showed relatively high serum gremlin-1 concentrations greater than our cutoff value 10.4 ng/mL. In contrast, compared with myofibroblasts and macrophages, we could not find apparent differences in the pattern of gremlin-1 protein expression in epithelial cells between IPF and non-IPF ILDs. Accordingly, the gap in serum gremlin-1 concentrations among patients with ILD appears to mainly reflect the total amount of myofibroblasts and perhaps that of M2-like macrophages.

A previous pathology study evaluated the utility of gremlin-1 for the differential diagnosis of ILD and reported that the mRNA and protein expression of gremlin-1 in lung samples was more increased in IPF than in NSIP [12], which is consistent with our results. However, the authors also showed that gremlin-1 expression assessed by lung immunostaining was negatively correlated with the patients’ pulmonary function, which was not apparent in our serum data. This might be explained by the difference in selected samples (lung specimens versus serum) and by a statistical limitation due to the relatively low number of participants in both studies. Although further verification with larger cohorts is essential, our data would broaden the possibility of gremlin-1 as a novel differential diagnosis biomarker using “serum”, which can be easily and non-invasively evaluated.

There have been numerous potential IPF biomarkers identified for evaluating disease severity and prognosis, including KL6, SP-A/D, matrix metalloproteinases, and osteopontin [8, 9, 24]. We recently reported that latent TGF-β binding protein-2 is a candidate for reflecting the process of fibroblast-to-myofibroblast differentiation [10]. However, biomarkers for the differential diagnosis between IPF and non-IPF ILDs are not frequently reported. Thus far, matrix metalloproteinases and their combination with other markers, such as SP-D and osteopontin, seem promising for this purpose [25, 26]. Although we could not directly compare these biomarkers with gremlin-1 in the same cohort, in spite of single molecule, the AUC value of gremlin-1 can be comparable to that of previous reports. To further increase the AUC value, new combinations of gremlin-1 with such candidate biomarkers would be worth trying.

In summary, gremlin-1 was upregulated in fibrotic lungs, particularly in IPF, and serum concentration measurements may be useful for improving the diagnostic certainty of IPF versus non-IPF ILDs. Our results again highlight the importance of gremlin-1 in the pathogenesis of IPF.

## Data Availability

Data can be provided by the corresponding author upon reasonable request.
